# Sophy β-Glucan from the Black Yeast *Aureobasidium pullulans* Attenuates *Salmonella*-Induced Intestinal Epithelial Barrier Injury in Caco-2 Cell Monolayers via Exerting Anti-Oxidant and Anti-Inflammatory Properties

**DOI:** 10.3390/antiox13010048

**Published:** 2023-12-28

**Authors:** Fangshen Guo, Hongbin Liu, Xiaomin Li, Zeqiong Hu, Jia Huang, Ruichen Bi, Waseem Abbas, Yuming Guo, Zhong Wang

**Affiliations:** State Key Laboratory of Animal Nutrition, College of Animal Science and Technology, China Agricultural University, Beijing 100193, China; b20203040314@cau.edu.cn (F.G.); s20233040782@cau.edu.cn (H.L.); s20213040650@cau.edu.cn (X.L.); s20203040626@cau.edu.cn (Z.H.); s20213040613@cau.edu.cn (J.H.); s20223040717@cau.edu.cn (R.B.); waseemabbas@cau.edu.cn (W.A.); guoyum@cau.edu.cn (Y.G.)

**Keywords:** sophy β-glucan, *Salmonella* Enteritidis, intestinal barrier function, immune, antioxidant, Caco-2 cells

## Abstract

The zoonotic pathogens *Salmonella* spp. infection disrupted intestinal epithelial barrier function and induced local gastroenteritis and systemic inflammation in humans and animals. Sophy β-glucan, a water-soluble β-1,3/1,6-glucan synthesized from the black yeast *Aureobasidium pullulans*, was reported with immune-regulatory, anti-inflammatory, and anti-infective properties. Here, we investigated the protective role of sophy β-glucan on *Salmonella enterica* serotype Enteritidis (SE)-challenged Caco-2 cells monolayer and explored underlying action mechanisms. The results showed that pretreatment with sophy β-glucan blocked the adhesion and invasion of SE onto Caco-2 cells along with alleviating SE-induced epithelial barrier injury, as evidenced by increased trans-epithelial electrical resistance, decreased fluorescently-labeled dextran 4 flux permeability, and an enhanced Claudin-4 protein level in the SE-stimulated Caco-2 cell monolayer. Moreover, treatment with β-glucan down-regulated pro-inflammatory factors (IL-1β, IL-8, and TNF-α) while up-regulating anti-inflammatory factors IL-10 at mRNA and protein levels in SE-infected Caco-2 cells. Furthermore, sophy β-glucan strengthened the anti-oxidative capacity of Caco-2 monolayers cells by elevating T-AOC and SOD activity and inhibiting MDA production defending SE. Together, our data showed that sophy β-glucan could prevent intestinal epithelial injury induced by SE, possibly by exerting anti-oxidant and anti-inflammatory properties, and it might be helpful for controlling SE infection.

## 1. Introduction

Intestinal epithelial cells (IECs) connect with each other in a continuous epithelial cell layer via an adhesive junctional proteins complex including tight junction (TJ) proteins, adherens junction proteins, gap junction proteins, and desmosomes, which contribute to the gut barrier integrity and function [1]. The intestinal epithelial barrier layer serves as a semipermeable structure that allows the absorption of nutrients and fluids simultaneously, and plays a crucial role in separating the host’s internal milieu from the external environment and preventing the transport of potentially harmful substances from entering the underlying tissue, including luminal microbes and antigenic molecules [2,3]. The disruption of intestinal barrier-related proteins due to adverse challenges could lead to impaired gut integrity, increased gut permeability, and gut inflammation [4,5]. Increased gut inflammation further perpetuates the disruption of TJ proteins, leading to increased permeability [6]. Thus, it is very necessary and particularly important to control intestinal disease by preventing TJ protein disruption and maintaining intestinal barrier integrity.

*Salmonella* spp., a gram-negative, intercellular, and food-borne pathogen, is able to induce diseases ranging from diarrhea, nausea, vomiting, fever, and abdominal pain to life-threatening systemic infections in humans after consuming contaminated water or animal and plant products [7]. *Salmonella enterica* serotype Enteritidis (SE) has become one of the most common serotypes reported in human salmonellosis worldwide, and leads to public health concern and huge economic loss [8,9,10]. SE infection in the gut is characterized by adhering and invading intestinal epithelial cells, inducing intestinal inflammatory responses, and impairing mucosal barrier functions by altering the tight junction protein, along with increased gut permeability, which leads to local gastroenteritis and systemic inflammation [11,12]. The treatments for salmonellosis are mainly by means of managing the inflammatory status with different drug categories, including antibiotics, probiotics, and immunosuppressants, which are inadequate and dangerous due to drug-resistant bacteria and drug residue [13]. However, natural antibiotics alternatives with anti-infective and barrier-protecting effects have received much attention to reduce the long-term antibiotics-induced risks and poor tolerability of the current drugs for treating salmonellosis in recent years [13,14].

Sophy β-glucan is a new type of exopolysaccharide synthesized from the black yeast *Aureobasidium pullulans* (*A. pullulans*) strain AFO-202 [15]. It is prepared by the Sophy Co. (Kochi, Japan) using the latest biological culture techniques (United States Patent 6,956,120 and Japan Patent 2004-329077). Structure analysis showed that sophy β-glucan is a water-soluble β-1,3/1,6-glucan, whose primary structural unit is a β-1,3-glucosidic-linked backbone with four β-1,6-glucosyl branching units every six residues [16,17,18]. In vitro and in vivo studies have demonstrated that sophy β-glucan from *A. pullulans* acts as a biological response modifier, including immune-regulatory, anti-inflammatory, anti-bacterial and viral infective, anti-oxidant, and anti-aging activities as well as accelerating wound healing [19,20,21]. Moreover, gut health-promoting and prebiotic-like functions of sophy β-glucan have been demonstrated in human and animal models [22]. Thus, sophy β-glucan from the black yeast *A. pullulans* is currently attracting attention due to its high potential for application as a health food supplement and biomedicine.

However, to date, the protective effects of sophy β-glucan on *Salmonella*-induced intestinal epithelial barrier injury and the underlying mechanism have not been investigated yet. In the present study, we investigated the protective capacity and underlying action mechanisms of sophy β-glucan on the *Salmonella*-stimulated TJ destruction of Caco-2 monolayer cells in vitro by assessing the epithelial barrier integrity, permeability, and immune and oxidative function, aiming to provide a novel approach for the treatment of *Salmonella* infection.

## 2. Materials and Methods

### 2.1. Materials and Reagents

Caco-2 cells (American Type Culture Collection, Rockville, MD, USA) were acquired from the National Collection of Authenticated Cell Cultures (Shanghai, China). Dulbecco’s modified eagle’s medium (DMEM), penicillin, streptomycin, gentamicin, trypsin-EDTA, and phenolsulfonphthalein were obtained from M&C gene Technology Co., Ltd. (Beijing, China). Transwell cell culture chamber inserts were obtained from Corning (Corning, NY, USA). Fluorescein isothiocyanate-dextran 4 (FITC-dextran) was purchased from Sigma-Aldrich (St. Louis, MO, USA). Kits for assaying cytokines were purchased from Boster Biological Technologies Co., Ltd., (Wuhan, China). Fetal bovine serum (FBS) was purchased from Gibco (Grand Island, NY, USA). RIPA lysis buffer, phenylmethanesulfonylfluoride (PMSF), BCA protein assay kit, SDS-PAGE gel preparation kit, and SDS-PAGE protein sample loading buffer were purchased from Beyotime Biotechnology (Shanghai, China). Polyvinylidene fluoride (PVDF) membranes were purchased from Millipore (Burlington, MA, USA). The antibodies for western-blot analysis including Claudin-1 (sc-166338, 22 kDa), Claudin-4 (sc-376643, 25 kDa), Occludin (sc-271842, 60–82 kDa), and Zo-1 (sc-33725, 220 kDa) were purchased from Santa Cruz Biotechnology, Inc. (Santa Cruz, CA, USA). The PrimeScript RT Reagent Kit with gDNA Eraser plus reagent and the SYBR Premix Ex Taq II kit were obtained from Takara Biotechnology Co., Ltd. (Dalian, China). Superoxide dismutase (SOD), glutathione (GSH), total antioxidant capacity (T-AOC), and malondialdehyde (MDA) detection kits were acquired from Nanjing Jiancheng Bioengineering Institute (Nanjing, China). Unless noted, all other reagents were obtained from Beyotime Biotechnology (Shanghai, China).

### 2.2. Caco-2 Cells Culture

Caco-2 cells were cultured in DMEM containing fetal bovine serum (10%), penicillin (100 U/mL), and streptomycin (100 μg/mL) in a humidified atmosphere at 37 °C, and 5% CO_2_ was passaged every 2 days. Subsequently, cells were sub-cultured at 85–90% confluence and differentiated into enterocyte-like cells, and then washed 3 times with DMEM basal medium for subsequent trials. 

### 2.3. Cell Viability

Cell viability was detected by 3-(4,5-dimethyl thiazol-2-yl)-2,5-diphenyl tetrazolium bromide (MTT) reduction assay [23]. Briefly, Caco-2 cells at a density of 5 × 10^5^ cells were treated with different concentrations of sophy β-glucan (0, 6.25, 12.5, 25, 50, 100, and 200 μg/mL) and LPS (50 μg/mL) for 24 h. Then, 10 μL of MTT solution (5 mg/mL, Beyotime, Nanjing, China) was added and further incubated for 2 h at 37 °C. Finally, after the removal of the supernatant, 100 µL of dimethyl sulfoxide was added to measure the absorbance at 450 nm. Cell viability was expressed as the percent of untreated cells.

### 2.4. Sophy β-Glucan Pre-Treatment, Salmonella Infection In Vitro and Samples Collection

SE CICC21513 was cultured on LB medium (Beijing Land Bridge Technology, Co., Ltd., Beijing, China). Fresh SE cultures were washed with PBS, counted, and then adjusted with DMEM basal medium (HyClone Laboratories, Logan, UT, USA) without FBS to 10^7^ CFU/mL for the next experiments. 

After changing the culture medium, Caco-2 cells were pre-treated with either sophy β-glucan or vehicle (MEM medium with 10% FBS without antibiotics). After inoculation for 24 h, half of the glucan-treated wells and half of the untreated wells were infected with SE (MOI = 100:1) and further incubated for another 3 h, while the remaining wells were only treated with antibiotics-free DMEM basal medium. Cells were removed using a cell scraper and collected in 1.5 mL tubes. Thereafter, after centrifugation at 4000× *g* for 5 min at 4 °C, the culture supernatant was collected for the detection of antioxidant and cytokines content, and cell precipitate was collected for the assay of cytokines’ mRNA, protein expression, and intracellular antioxidant enzymes activities. All samples were assessed in triplicate. 

### 2.5. Bacterial Adhesion and Invasion Assay

The adhesion and invasion ability assay of *Salmonella* to cells was performed as previously reported [24]. Briefly, after 2 h of SE incubation, cell cultures were washed twice with PBS and lysed with trypsin-EDTA (Invitrogen, Waltham, MA, USA; Life Technologies, Canton, OH, USA) for 10 min at 37 °C. Samples were collected and diluted using serial PBS. *Salmonella* counts were conducted using the plate method onto *Salmonella* selective XLT Agar at 37 °C for 24 h. Invaded bacteria were acquired by adding DMEM containing 150 μg/mL gentamicin (Sigma-Aldrich Chemie GmbH, Buchs, Switzerland) and incubated at 37 °C for 60 min. After washing by PBS, 250 μL of trypsin-EDTA (Life Technologies, OH, USA) was added for the incubation for 10 min at 37 °C. Cells were then disrupted by adding 250 μL of Triton X-100 (0.1%, Sigma, St. Louis, MO, USA). After 10 min of incubation, the samples were collected to determine the *Salmonella* counts. 

### 2.6. Measurement of Trans-Epithelial Electrical Resistance (TER) and Permeability

To determine the effects of sophy β-glucan on the TJ stabilization of Caco-2 cells, TER assay was performed as described in a previous study, although with modifications [25]. Caco-2 cells (5 × 10^5^ cells/cm^2^) were seeded in the upper chamber of 12-well Trans-well^®^ plates (Corning Costar, Corning, NY, USA) on polyester membrane filters (pore size 0.4 µm, surface area 1.12 cm^2^). Culture medium was added to both the apical and basal chamber and the medium was changed every other day until complete differentiation into small intestinal-like epithelial cells. For the Caco-2 cells monolayer, the TER was measured and calculated using the EMD Millipore Millicell-ERS2 Volt-Ohm Meter (Merck-Millipore, Burlington, VT, USA). Filters (with a cell monolayer) showing more than 500 Ωcm^2^ were used for a permeability study. Following cells were pretreated with vehicle (DEME basal medium alone, as controls) and sophy β-glucan (final concentration of 50 μg/mL, dissolved in DEME basal medium with FBS but no antibiotics), which were added to the upper compartment for 24 h, respectively. After treatment, the monolayer was washed with PBS to remove residual glucan, and half of the glucan-treated and untreated wells was further stimulated with vehicle (DEME basal medium without FBS and antibiotics) and 20 μL of SE (MOI = 100:1, dissolved in DEME basal medium), respectively. The TER was measured before and at 0.5, 1, 3, and 6 h after stimulation with SE. Triplet cell monolayers were assessed for each experimental group. The TER values during the experimental conditions were calculated and expressed as the percentage relative to the control group.

Meanwhile, the permeability of FITC-dextran (Sigma Aldrich, USA) across the Caco-2 cell monolayer was measured as previously described with modifications [26]. First, 200 μL of FITC-dextran solution (5 mg/mL) and 500 µL of PBS were added to the apical and basolateral compartments of each trans-well insert, respectively, after the cell monolayer was treated with SE, and then washed with PBS twice. Then, 100 µL of the sample in the basolateral compartment was taken and transferred to 96-well plates at 1, 3, 6, and 9 h of incubation at 37 °C, respectively. Subsequently, FITC-dextran concentration was determined at an excitation wavelength of 492 and an emission wavelength of 520 nm, respectively. All samples were assessed three times.

### 2.7. Supernatants Cytokines

The amount of cytokines IL-1β, IL-8, TNF-α, and IL-10 in culture supernatants was determined using cytokines commercial kits (Boster Biological Technologies Co., Ltd.), according to the manufacturer’s instructions.

### 2.8. Antioxidant Indices

Cell precipitate was lysed with 150 μL of RIPA lysis buffer (Beyotime Biotechnology, Shanghai, China) and cell lysates were then collected through centrifugation (12,000× *g*, 15 min, 4 °C). SOD, GSH, T-AOC, malondialdehyde (MDA), and GSH-Px activities in cell supernatant and cell lysates were determined by using Detection Kits (Nanjing Jiancheng Bioengineering Institute, Nanjing, China), respectively.

### 2.9. Quantitative Real-Time PCR Analysis

The total RNA of Caco-2 cells was extracted using the RNAeasy Mini kit (Qiagen, San Jose, CA, USA) according to the manufacturer’s protocol. The quantity and quality of the extracted total RNA were determined using a spectrophotometer (NanoDrop-2000; Thermo Fisher Scientific, Waltham, MA, USA) at 260 and 280 nm. Next, RNA was reverse-transcribed into cDNA using the PrimeScript RT Reagent Kit with a gDNA Eraser (Takara Bio, Kusatsu, Japan), according to the manufacturer’s instructions. Real-time PCR for monitoring the mRNA expression of inflammatory cytokines and tight junction proteins was carried out using SYBR Premix Ex Taq II (Takara, Otsu, Japan) and measured by the 7500 quantitative PCR System (Stratagene, San Diego, CA, USA). The sequences of the primers used for RT-qPCR are provided in Table 1.

### 2.10. Western Blotting

Caco-2 cells were lysed with 150 μL of RIPA lysis buffer containing 10 μL of PMSF (Beyotime Biotechnology, Shanghai, China) for the extraction of total proteins through centrifugation (12,000× *g*, 15 min, 4 °C). Protein concentration was determined using the BCA protein assay kit (Beyotime Biotechnology, Shanghai, China). The loading buffer was added to the protein samples, and the mixture was boiled for 10 min. The mixture containing equal amounts of protein (50 μg) from each sample was loaded to 8%, 10%, or 12% SDS-PAGE gels to separate the proteins before being transferred to a polyvinylidene difluoride (PVDF) membrane. The membrane was incubated with 5% skim milk at room temperature for 2 h, and then incubated with the diluted primary antibodies including Claudin-1, Claudin-4, Occludin, and Zo-1 (1:1000 dilution, Santa Cruz Biotechnology. Inc.) at 4 °C overnight. After being washed twice with Tris buffered saline with Tween 20 (TBST), the membranes were incubated with the corresponding HRP-conjugated secondary antibody (diluted, 1:5000) for 1 h at room temperature. Thereafter, the protein bands of the membrane were visualized by using a super enhanced chemiluminescence (ECL) plus detection system (P0018, Beyotime, China) and documented with X-ray film (Clinx Science Instruments Co., Ltd., Shanghai, China). Images were captured using an imaging system (Bio-Rad Laboratories, Inc., Hercules, CA, USA). The protein levels were analyzed with Image J Software (https://imagej.net/ij/download.html, accessed on 22 November 2023, National Institutes of Health, Bethesda, MD, USA). β-actin protein expression was used as an internal control. Data processing was completed using Quantity One software Version 4.6.3.

### 2.11. Statistical Analyses

The SPSS 20.0 software for Windows was used for all statistical analyses in this study. The adhesion power, invasion power of SE and TER, FITC-dextran diffusion, and WB results were analyzed using *t*-test. Univariate analysis was used to analyze the main effect and interactive effect of sophy β-glucan and SE on genes as well as oxidative indexes. All data are expressed as the Mean ± SEM, and *p* < 0.05 was implied as a significant difference. Plots were performed using GraphPad Prism 8.0.2.

## 3. Results

### 3.1. Effect of Sophy β-Glucan on the Cell Viability of Caco-2 Cells

**Figure 1:**
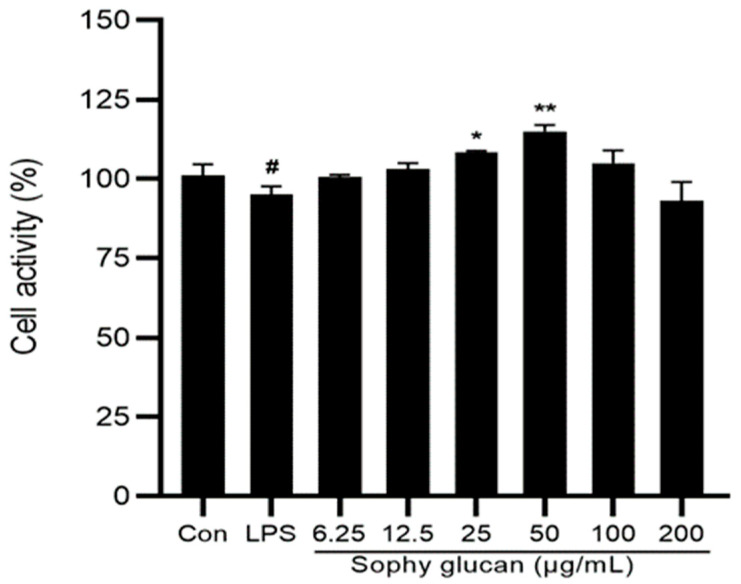
Cytotoxic effects of sophy β-glucan on Caco-2 cells’ viability. Indicated concentrations of sophy β-glucan and LPS (50 μg/mL) were used for cell treatment for 24 h. Values are shown as means ± SEM. # 0.05 < *p* < 0.10, * 0.01 < *p* < 0.05, ** 0.001 < *p* < 0.01 compared with untreated control cells.

MTT assay was performed for the determination of optimal concentrations of sophy β-glucan on Caco-2 cells’ viability. As shown in Figure 1, LPS treatment trended to reduce the cell viability of Caco-2 cells compared with the untreated negative control (0.05 < *p* < 0.1). However, cell viability was practically unaffected after the incubation of Caco-2 cells with 6.25, 12.5, 100, and 200 μg/mL of sophy β-glucan for 24 h as compared to the untreated cells, with all cell survival rates remaining above 95% (*p* > 0.05), suggesting that sophy β-glucan had no cytotoxicity to Caco-2 cells after 24 h at a concentration of less than 200 μg/mL. The incubation of Caco-2 cells with sophy β-glucan at 25 and 50 μg/mL for 24 h resulted in a significant increase in cell viability in a dose-dependent manner (*p* < 0.05). Thus, 50 μg/mL were chosen as the optimal concentrations for further experiments.

### 3.2. Sophy β-Glucan Reduced the Adhesion and Invasion of SE into the Caco-2 Cell Monolayer

**Figure 2:**
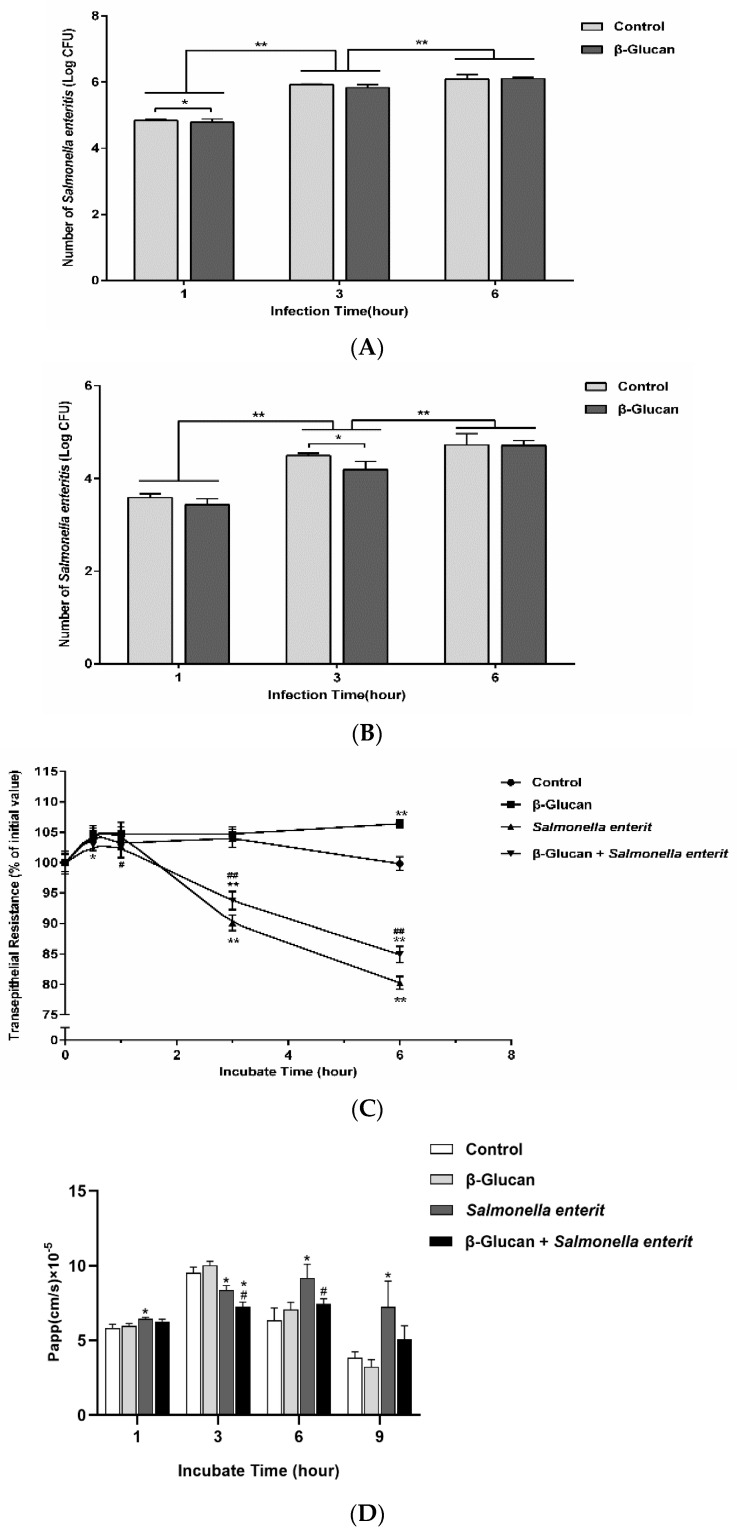
Sophy β-glucan improved gut permeability in *Salmonella*-stimulated Caco-2 cell monolayer. (**A**) β-glucan inhibited the adhesion and (**B**) invasion power of *Salmonella* onto cells. (**C**) β-glucan strengthened the TER. (**D**) β-glucan held flux of fluorescein isothiocyanate dextran 4 across monolayers. Data are shown as the means ± SEM, * *p* < 0.05, ** *p* < 0.01 compared with control group. # *p* < 0.05, ## *p* < 0.01 compared with *Salmonella* Enteritidis-challenged group.

To determine the protective role of sophy β-glucan on gut epithelial cells, the adhesion and invasion activity of SE onto the Caco-2 cell monolayer was performed. As expected, when the infection time increased, the adhesion and invasion of SE to the Caco-2 cell monolayer was remarkably elevated in the absence of sophy β-glucan (Figure 2A,B, *p* < 0.01). However, sophy β-glucan pretreatment notably reduced the adhesion and invasion power of *Salmonella* to the Caco-2 cells monolayer at 1 and 3 h post infection (hpi), respectively (*p* < 0.05).

### 3.3. Sophy β-Glucan Ameliorates the Intestinal Epithelial Barrier Dysfunction Induced by SE

The intestinal barrier plays a potent role in hindering SE invasion into enterocyte and translocation over gut. TER and cell monolayer FITC-dextran permeability experiments were performed to assess the intestinal barrier function. In this study, we firstly evaluated the protective effects of sophy β-glucan on the intestinal epithelial barrier function of polarized Caco-2 cell monolayers disrupted by SE through measuring the TER (Figure 2C). As a result, SE infection induced a significant increase in the permeabilization of the Caco-2 monolayers, as shown by decreased TER values at 3 and 6 h post-infection compared with the negative control (*p* < 0.05), indicating that SE infection destroyed the barrier functions of the Caco-2 cell monolayer. By contrast, pretreatment with sophy β-glucan significantly reversed the detrimental impact of the TER caused by the SE challenge at 6 hpi (*p* < 0.01).

Moreover, another appearance of Caco-2 monolayer permeability FITC-dextran flux was measured (Figure 2D). Consistent with the TER results, the SE infection increased FITC-dextran diffusion over the Caco-2 cell monolayer at 1, 6, and 9 hpi (*p* < 0.05). However, pretreatment with sophy β-glucan recovered this “leaky gut” phenomenon at 3 and 6 hpi of incubation (*p* < 0.05). Together, these findings suggested that sophy β-glucan could exert a protective effect on SE-induced paracellular permeability in intestinal epithelial cells under a SE challenge.

### 3.4. Sophy β-Glucan Prevents SE-Induced Disruption of TJ in Caco-2 Cell Monolayer

**Figure 3:**
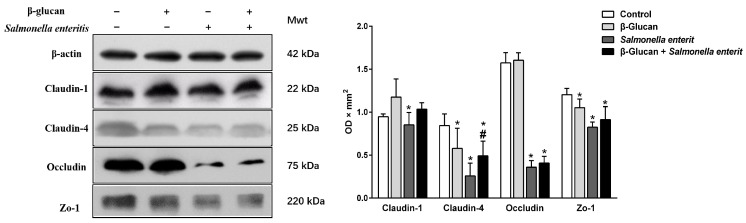
Sophy β-glucan advanced tight junction expression in *Salmonella*-stimulated Caco-2 cell monolayer. Data are shown as the means ± SEM, * *p* < 0.05 compared with control group. # *p* < 0.05 compared with *Salmonella* Enteritidis-challenged group.

TJ was involved in the formation, function, and integrity of the intestinal barrier. Thus, the expression levels of TJ-associated factors were investigated by RT-qPCR and western blot analyses in order to determine the mechanisms underlying the protective effect of sophy β-glucan on epithelial cell monolayer permeability disrupted by *Salmonella* in the Caco-2 cell. The results of RT-qPCR revealed that the mRNA expression levels of Claudin-1, Caudin-4, and Zo-1 in the Caco-2 cell were significantly up-regulated after SE treatment alone compared with those in the non-infected control group (Table 2, *p* < 0.05). Also, Caco-2 cells treated with sophy β-glucan showed significant up-regulation in Zo-1, Occludin, and Claudin-4 mRNA levels compared with the non-treated and uninfected control (*p* < 0.05). Surprisingly, pretreatment with sophy β-glucan significantly down-regulated Occludin, Caludin-1, and Claudin-4 mRNA levels in *Salmonella*-induced Caco-2 cells at 3 hpi (*p* < 0.05). Interestingly, western blotting results observed that, compared with the control group, the SE challenge diminished the protein expression levels of Claudin-1, Claudin-4, Occludin, and Zo-1 in Caco-2 cells (*p* < 0.05). However, the pre-administration of sophy β-glucan notably restored the Claudin-4 protein amount in *Salmonella*-induced Caco-2 cells, which were still lower than the control group (*p* < 0.05, Figure 3). These observations suggested that preventing the reduced expression levels of the TJ protein Caludin-4 may be the mechanism underlying the effects of sophy β-glucan on the *Salmonella*-induced increase in epithelial cell monolayer permeability.

### 3.5. Sophy β-Glucan Suppressed the SE-Induced Pro-Inflammatory Status of the Caco-2 Cell

Cytokines play an essential role in modulating the inflammatory response and immune status together with the inflammation-induced disruption of the barrier function. Therefore, the mRNA and protein levels of the released cytokines were analyzed. As illustrated in Table 2, SE infection significantly induced up-regulated mRNA expression of IL-1β, IL-8, and IL-10, as well as TNF-α mRNA levels (*p* < 0.05), suggesting that SE infection induced inflammatory responses in Caco-2 cells. Analogously, sophy β-glucan strongly induced up-regulation in IL-1β and IL-10 mRNA of Caco-2 cells (*p* < 0.05). However, decreased mRNA levels of pro-inflammatory factors IL-1β, IL-8, and TNF-α in cell monolayers were significantly detected when sophy β-glucan was given under the SE challenge, while anti-inflammatory factors IL-10 and TGF-β mRNA were up-regulated (*p* < 0.05). Similarly, increased levels of IL-1β, IL-8, and TNF-α secreted by the Caco-2 cells into the cell supernatant induced by SE infection were observed when compared with the negative control, while anti-inflammatory cytokines IL-10 decreased (Table 3, *p* < 0.05). The Caco-2 cells stimulated with sophy β-glucan also strongly induced IL-1β, IL-8, and IL-10 production in the absence of a challenge (*p* < 0.05), indicating that sophy β-glucan possesses immune-stimulatory activity. However, as compared with the SE challenge group, the Caco-2 cells pretreated with sophy β-glucan showed a notable reduction in TNF-α production and an increase in the IL-1β, IL-8, and IL-10 amount in the cell supernatants (*p* < 0.01). These results indicated that sophy β-glucan can regulate the *Salmonella*-induced production of pro-inflammatory and anti-inflammatory cytokines in Caco-2 cell models.

### 3.6. Sophy β-Glucan Enhanced Anti-Oxidative Functions in SE-Stimulated Caco-2 Cells

To deeply reveal the protective efficacy and underlying action mechanism of sophy β-glucan on SE-induced intestinal epithelial barrier injury, we further evaluated the effect of sophy β-glucan on anti-oxidative functions in SE-stimulated Caco-2 cells. As revealed in Table 3, compared with unstimulated cells, SE infection significantly increased the T-AOC and GSH activity in the cell supernatant at 3 hpi, and MDA content was also raised at 12 h after *Salmonella* infection alone (*p* < 0.05). Different from the above observation, the exposure of the cells to SE infection alone notably decreased intracellular SOD (3 hpi) in the cell lysates (*p* < 0.05). Compared with unstimulated cells, Caco-2 cells treated with sophy β-glucan alone showed a significant increase in T-AOC levels (3 hpi) in the cell supernatant and intercellular SOD (3 hpi) and GSH activity (12 hpi, *p* < 0.05), while GSH activity in the cell supernatant was decreased by sophy β-glucan treatment alone in Caco-2 cells (*p* < 0.05). Sophy β-glucan pretreatment remarkably increased T-AOC content (at 3 and 12 hpi) in SE-infected cells and intercellular SOD activity (3 hpi), but significantly decreased MDA levels in the cell supernatant at 12 hpi (*p* < 0.05).

## 4. Discussion

This study investigated the barrier-protecting effect of sophy β-glucan in *Salmonella*-stimulated Caco-2 monolayer cells and further explored its underlying action mechanisms. Adhesion and invasion by *Salmonella* could orchestrate inflammatory reaction through delivering *Salmonella* pathogenicity island virulent proteins into host cell cytoplasm, which will lead to the alteration of the intestinal epithelial barrier function [27]. In this study, we first evaluated the effects of sophy β-glucan pretreatment on the adhesion and invasion abilities of SE onto Caco-2 cells. Our data showed that sophy β-glucan pretreatment notably reduced the adhesion and invasion power of *Salmonella* onto the intestine-like cell monolayer line. Similarly, a previous study had confirmed the suppressed adhesion and invasion of SE to the intestine induced by β-glucan through competitively attaching epithelial binding sites or agglutinating with *Salmonella*, thus presumably reducing the adhesion capacity of *Salmonella* to the surface of intestinal epithelial cells [28]. Additionally, we observed that SE infection damaged intestinal epithelial barrier integrity and increased permeability, as evidenced by decreased TER values and an increased FITC-dextran influx. Interestingly, the results of RT-qPCR found that the SE challenge up-regulated Claudin-1, Claudin-4, and Zo-1 mRNA expressions in Caco-2 cells, but induced a notable reduction in the protein expression levels of TJ-associated factors, Claudin-1, Claudin-4, Occludin, and Zo-1. The reason for the asynchronism of the transcription and translation of tight junction proteins are possibly due to the post-translational modification of genes. The down-regulated TJs proteins abundance obtained in our study showed that SE infection damaged intestinal epithelial barrier integrity by decreasing TJ proteins’ expression level in Caco-2 cells. Conversely, these changes induced by SE infection were partially abolished by sophy β-glucan pre-treatment. Namely, Caco-2 cells pre-incubated with sophy β-glucan notably up-regulated the Claudin-4 protein amount in *Salmonella*-infected Caco-2 cells. This result matched the consequence of TER and FITC-dextran influx. In similarity with our findings, a study has shown that the pre-administration of yeast β-glucan was able to protect intestinal epithelial barrier integrity by increasing the expressions levels of TJ in *Salmonella*-infected birds [29]. Thus, our data suggested that pre-treatment with water-soluble sophy β-glucan could contribute to maintaining the integrity of the epithelial barrier under the SE model, partially through reducing *Salmonella* adhesion and invasion onto intestinal epithelial cells and up-regulating Claudin-4 protein abundance.

Pro-inflammatory cytokines such as TNF-α, interferon-γ, and IL-1β have been recognized to contribute to intestinal barrier dysfunction and exacerbate inflammatory responses, while anti-inflammatory cytokines (IL-10 and TGF-β) have been reported to be positively associated with intestinal barrier tissue repair during the course of inflammatory bowel disease [30]. In this study, the interaction of SE with intestinal epithelial cells induced obvious inflammatory responses in Caco-2 cells, as proven by up-regulating pro-inflammatory cytokines (IL-1β, IL-8, and TNF-α), which was consistent with the results of previous studies [31,32,33,34]. Interestingly, SE boosted IL-10 mRNA levels but decreased IL-10 protein levels. As we know, mRNA transcription and protein translation are not always consistent at the same sampling time-point; thus, the inconsistence between the IL-10 mRNA level and protein amount was possibly associated with post-transcription regulation. But protein was the functionally biological molecule. Furthermore, our data suggested that *Salmonella* Enteritidis infection damaged intestinal epithelial barrier function, possibly through promoting the overexpression of pro-inflammatory cytokines. Similarly, the administration of sophy β-glucan notably induced up-regulation in IL-1β and IL-10 at both mRNA and protein levels secretion of non-infected Caco-2 cells, but without impairment on epithelial cell barrier function in our study, which was in agreement with previous results in PBMCs, bone marrow-derived DCs, splenocytes, and macrophages [35,36,37,38,39,40], showing that sophy β-glucan could enhance the immune function of intestinal epithelial cells under non-challenged conditions. Additionally, *Salmonella*-induced elevated TNF-α synthesis and secretion and down-regulated IL-10 by Caco-2 cells, which was reversed by sophy β-glucan, indicating that sophy β-glucan possessed anti-inflammatory properties against *Salmonella*-induced inflammatory responses in intestinal epithelial cells. Interestingly, the variation trends of pro-inflammatory cytokines IL-1β and IL-8 were not the same as TNF-α content. Cytokines IL-1β and IL-8 were reported to be involved in the restriction of *Salmonella* infection [41], whereas TNF-α was shown to be elevated in the intestinal mucosa of inflammatory bowel disease patients, which can lead to excess inflammation and gut injury [42,43]. Our observation also indicated that sophy β-glucan might restrain SE-induced intestinal inflammation mainly via inhibiting TNF-α expression, not IL-1β or IL-8. This phenomenon may account for a delicate regulation between the inflammation response and *Salmonella* obliteration of sophy β-glucan on Caco-2 cell under the SE challenge. Similarly, previous studies have demonstrated that the oral administration of soluble exopolysaccharide β-1,3/1,6-glucan from a black yeast, *A. pullulans*-cultured fluid could alleviate inflammatory responses by modulating immune cells’ activity and attenuating the expression of these pro-inflammatory cytokines in vivo and in vitro under an inflammatory stimulus [19,44,45]. Thus, our findings suggested that sophy β-glucan might be an effective immune modulator and a potential anti-inflammatory agent for the treatment of salmonellosis. These results also explained that the protective role of sophy β-glucan on intestinal epithelial barrier function was possibly associated with its anti-inflammatory ability.

Oxidative stress is associated with intestinal inflammation and disease progress [46]. Oxidative stress is also considered one of the main factors involved in epithelial barrier function disruption [47]. The development of new means of protecting the gut from oxidative stress and inflammatory cytokines during the progression of intestinal diseases may offer novel therapeutic strategies to mitigate intestinal barrier injury. Therefore, we investigated the effect of sophy β-glucan on the intracellular and extracellular redox status of Caco-2 cells stimulated with SE or not. Surprisingly, SE infection alone significantly raised the T-AOC, GSH activity in the cell supernatant both at the early and late stages of infection, but decreased intracellular SOD and GSH activities. These alterations may be attributed to the active defense against the oxidative damage of intestinal epithelial cells after *Salmonella* infection [48]. Additionally, our data also found that SE infection increased MDA levels at the later phase of infection. The end-product of lipid peroxidation, MDA, is a sensitive index assessing the degree of oxidative injury of the cell membrane, which was linked to redox imbalance, chronic inflammation, and mucosal barrier impairment in the gut [49]. Elevated MDA in SE-stimulated cells indicated that SE infection caused an imbalance of the redox status, resulting in oxidative stress and inflammatory responses of intestinal epithelial cells as well as the disruption of intestinal epithelial barrier function. On the other hand, we observed that Caco-2 cells treated with sophy β-glucan alone showed a significant increase in T-AOC levels (3 hpi) in the cell supernatant, as well as intercellular SOD (3 hpi) and GSH (12 hpi) activities, and a notable decrease in GSH activity (3 and 12 hpi) in the cell supernatant, compared with non-stimulated cells. The increases in T-AOC, GSH, and SOD in Caco-2 cells implied that sophy β-glucan could activate the anti-oxidative status in intestinal epithelial cells either directly or indirectly, which agreed with previous observations [50]. However, in the presence of *Salmonella* infection, the cells that underwent pretreatment with sophy β-glucan displayed a remarkable increase in T-AOC (3 and 12 hpi) and SOD activity in the cell supernatant and SOD activity in the cell lysates, and a notable reduction in MDA concentration in the cell supernatant, showing that sophy β-glucan could enhance the anti-oxidant ability of intestinal epithelial cells against the oxidative stress induced by *Salmonella* infection. This finding also meant that the protective effect of sophy β-glucan on intestinal barrier function was possibly associated with its anti-oxidant functions.

## 5. Conclusions

In conclusion, our data suggested that sophy β-glucan pretreatment could attenuate the intestinal epithelial barrier impairment triggered by *Salmonella enterica* serotype Enteritidis infection in the Caco-2 cell monolayer, possibly by reducing *Salmonella* adhesion and invasion, suppressing the production of pro-inflammatory cytokines, modulating anti-oxidative functions, as well as strengthening the expression of the tight junction protein Claudin-4 level in intestinal epithelial cells. Overall, these data showed that sophy β-glucan might be useful as an effective strategy for preventing or treating intestine cell damage in salmonellosis and defending invasive salmonellosis.

## Figures and Tables

**Table 1:** Real-time PCR primers.

Target Genes	Primer Sequence (5′~3′)	Accession NO.
GAPDH	F: GCACCGTCAAGGCTGAGAAC	NM_002046.7
	R: ATGGTGGTGAAGACGCCAGT	
TNF-α	F: TAGCCCATGTTGTAGCAAACC	NM_000594.3
	R: ATGAGGTACAGGCCCTCTGAT	
IL-1β	F: AGTGGCAATGAGGATGACTTGT	NM_000576.2
	R: AGATGAAGGGAAAGAAGGTGCT	
IL-8	F: TTGCCAAGGAGTGCTAAAGAA	NM_000584.3
	R: GCCCTCTTCAAAAACTTCTCC	
IL-10	F: TTTAAGGGTTACCTGGGTTGC	NM_000572.2
	R: TTGATGTCTGGGTCTTGGTTC	
TGF-β	F: GCCAGAGTGGTTATCTTTTGATG	NM_00060.4
	R: AGTGTGTTATCCCTGCTGTCAC	
Claudin-1	F: AGAAGATGAGGATGGCTGTCA	NM_021101.4
	R: TTGGTGTTGGGTAAGAGGTTG	
Claudin-2	F: TTCTTCCCTGTTCTCCCTGAT	NM_020384.3
	R: CCCCTGGTTCTTCACACATAC	
Claudin-4	F: TATGGATGAACTGCGTGGTG	NM_001305.3
	R: CACGATGAACTGCGTGGTG	
Occludin	F: CCTTCACCCCCATCTGACTAT	NM_002538
	R: CTTTGACCTTCCTGCTCTTCC	
Zo-1	F: GGATGTTTATCGTCGCATTGTA	NM_003257.3
	R: AAGAGCCCAGTTTTCCATTGTA	

F, forward; R, reverse; Primers were synthesized by Sango Biotech (Shanghai, China) Co., Ltd.; GAPDH, glyceraldehyde-3-phosphate dehydrogenase; TNF-α, tumor necrosis factor-α; IL, interleukin; TGF-β, transforming growth factor-β; Zo-1, zonula occludens-1.

**Table 2:** Sophy β-glucan modulated the tight junction proteins, immune-related genes expression, and cytokines levels in the supernatant in *Salmonella*-stimulated Caco-2 cell monolayer.

Items	Sophy β-Glucan Levels, μg/mL				SEM	*p*-Values		
	0		50					
	Saline	SE	Saline	SE		β-glucan	SE	Interaction
Tight junction protein genes expression								
Claudin-1	1.00 ^b^	1.49 ^a^	1.00 ^b^	1.00 ^b^	0.023	0.017	0.012	0.001
Claudin-4	1.00 ^c^	2.19 ^a^	1.24 ^b^	1.10 ^bc^	0.052	0.001	0.001	0.001
Occludin	1.00 ^cd^	1.01 ^bc^	1.55 ^a^	0.74 ^d^	0.056	0.030	0.001	0.001
Zo-1	1.00 ^d^	1.29 ^bc^	1.48 ^a^	1.23 ^cd^	0.049	0.670	0.579	0.001
Pro-inflammatory-related genes expressions								
IL-1β	1.00 ^c^	5.62 ^a^	3.60 ^b^	1.08 ^c^	0.860	0.001	0.001	0.010
IL-8	1.00 ^c^	7.08 ^a^	1.24 ^c^	4.53 ^b^	0.079	0.001	0.001	0.001
TNF-α	1.00 ^c^	6.93 ^a^	1.44 ^c^	4.88 ^b^	0.243	0.005	0.001	0.001
Anti-inflammatory-related genes								
IL-10	1.00 ^c^	3.48 ^b^	3.67 ^b^	9.84 ^a^	0.244	0.001	0.001	0.001
TGF-β	1.00 ^ab^	0.67 ^cd^	0.85 ^bc^	1.08 ^a^	0.073	0.106	0.513	0.001
Pro-inflammatory cytokines levels								
IL-1β (pg/mL)	8.56 ^d^	62.8 ^b^	45.5 ^c^	82.4 ^a^	2.48	0.001	<0.001	0.001
IL-8 (pg/mL)	36.8 ^d^	259.6 ^b^	98.1 ^c^	339.6 ^a^	10.6	<0.001	<0.001	0.001
TNF-α (pg/mL)	32.7 ^c^	114.5 ^a^	49.6 ^c^	72.9 ^b^	11.5	0.002	<0.001	0.004
Anti-inflammatory-cytokines level								
IL-10 (pg/mL)	45.3 ^b^	23.5 ^c^	106.9 ^a^	148.3 ^a^	4.54	<0.001	0.033	0.002

Data are presented as mean ± SEM (*n* = 3); means within a row lacking a common superscript differ significantly (*p* < 0.05); SEM, standard error mean; SE, *Salmonella* Enteritidis; Zo-1, zonula occludens-1; IL, interleukin; TNF-α, tumor necrosis factor-α; TGF-β, transforming growth factor-β.

**Table 3:** Sophy β-glucan enhanced anti-oxidative capacity in *Salmonella*-stimulated Caco-2 cell monolayer.

Items	Sophy β-Glucan Levels, μg/mL				SEM	*p*-Values		
	0		50					
	Saline	SE	Saline	SE		β-Glucan	SE	Interaction
Cell culture supernatant 3 h post-infection								
MDA (nmol/mL)	0.53	0.45	0.45	0.31	0.025	0.002	0.002	0.256
T-AOC (U/mL)	0.44 ^c^	0.94 ^b^	1.42 ^a^	1.36 ^a^	0.122	0.001	0.019	0.006
SOD (U/mL)	28.51	44.26	32.86	47.47	2.366	0.001	0.001	0.189
GSH (U/mL)	17.21 ^b^	25.42 ^a^	12.12 ^c^	24.38 ^a^	1.661	0.002	0.001	0.017
12 h post-infection								
MDA (nmol/mL)	0.37 ^b^	0.61 ^a^	0.39 ^b^	0.35 ^b^	0.030	0.011	0.004	0.001
T-AOC (U/mL)	0.67 ^c^	1.14 ^b^	0.69 ^c^	1.95 ^a^	0.157	0.001	0.001	0.001
SOD (U/mL)	21.85	39.77	33.66	47.50	2.717	0.006	0.001	0.927
GSH (U/mL)	17.50 ^b^	20.33 ^a^	11.08 ^c^	21.08 ^a^	1.194	0.001	0.001	0.001
Cell lysates 3 h post-infection								
SOD (U/mgpro)	47.69 ^b^	43.82 ^c^	48.31 ^a^	46.51 ^b^	0.276	0.364	0.001	0.022
GSH (U/mg)	9.57	7.59	9.67	7.22	0.368	0.694	0.001	0.515
12 h post-infection								
SOD (U/mg)	42.86	26.23	45.49	29.93	2.518	0.001	0.001	0.927
GSH (U/mg)	9.20 ^cd^	9.48 ^bc^	10.61 ^ab^	8.16 ^d^	0.307	0.901	0.019	0.006

Data are presented as mean ± SEM (*n* = 3); means within a row lacking a common superscript differ significantly (*p* < 0.05); SEM, standard error mean; SE, *Salmonella* Enteritidis; MDA, malondialdehyde; T-AOC, total antioxidant capacity; SOD, superoxide dismutase; GSH, glutathione.

## Data Availability

The data presented in this study are available in the article.

## References

[1] Peterson L.W., Artis D. (2014). Intestinal epithelial cells: Regulators of barrier function and immune homeostasis. Nat. Rev. Immunol..

[2] Di Tommaso N., Gasbarrini A., Ponziani F.R. (2021). Intestinal barrier in human health and disease. Int. J. Environ. Res. Public Health.

[3] Artis D. (2008). Epithelial-cell recognition of commensal bacteria and maintenance of immune homeostasis in the gut. Nat. Rev. Immunol..

[4] Qin H., Zhang Z., Hang X., Jiang Y. (2009). *L. plantarum* prevents enteroinvasive Escherichia coli-induced tight junction proteins changes in intestinal epithelial cells. BMC Microbiol..

[5] Suzuki T. (2013). Regulation of intestinal epithelial permeability by tight junctions. Cell. Mol. Life Sci..

[6] Turner J.R. (2009). Intestinal mucosal barrier function in health and disease. Nat. Rev. Immunol..

[7] Eng S.-K., Pusparajah P., Ab Mutalib N.-S., Ser H.-L., Chan K.-G., Lee L.-H. (2015). *Salmonella*: A review on pathogenesis, epidemiology and antibiotic resistance. Front. Life Sci..

[8] Majowicz S.E., Musto J., Scallan E., Angulo F.J., Kirk M., O’Brien S.J., Jones T.F., Fazil A., Hoekstra R.M. (2010). The global burden of nontyphoidal *Salmonella* gastroenteritis. Clin. Infect. Dis..

[9] Koutsoumanis K., Allende A., Alvarez-Ordóñez A., Bolton D., Bover-Cid S., Chemaly M., De Cesare A., Herman L., Hilbert F., Lindqvist R. (2019). *Salmonella* control in poultry flocks and its public health impact. EFSA J..

[10] Balasubramanian R., Im J., Lee J.-S., Jeon H.J., Mogeni O.D., Kim J.H., Rakotozandrindrainy R., Baker S., Marks F. (2019). The global burden and epidemiology of invasive non-typhoidal *Salmonella* infections. Hum. Vaccin. Immunother..

[11] Rychlik I., Elsheimer-Matulova M., Kyrova K. (2014). Gene expression in the chicken caecum in response to infections with non-typhoid *Salmonella*. Vet. Res..

[12] Awad W.A., Hess C., Hess M. (2017). Enteric pathogens and their toxin-induced disruption of the intestinal barrier through alteration of tight junctions in chickens. Toxins.

[13] Mkangara M. (2023). Prevention and control of human *Salmonella enterica* infections: An implication in food safety. Int. J. Food Sci..

[14] Camilleri M. (2021). Human intestinal barrier: Effects of stressors, diet, prebiotics, and probiotics. Clin. Transl. Gastroenterol..

[15] Prasongsuk S., Lotrakul P., Ali I., Bankeeree W., Punnapayak H. (2018). The current status of *Aureobasidium pullulans* in biotechnology. Folia Microbiol..

[16] Kono H., Kondo N., Isono T., Ogata M., Hirabayashi K. (2020). Characterization of the secondary structure and order-disorder transition of a β-(1 → 3, 1 → 6)-glucan from *Aureobasidium pullulans*. Int. J. Biol. Macromol..

[17] Kono H., Kondo N., Hirabayashi K., Ogata M., Totani K., Ikematsu S., Osada M. (2017). NMR spectroscopic structural characterization of a water-soluble β-(1→3, 1→6)-glucan from *Aureobasidium pullulans*. Carbohydr. Polym..

[18] Muramatsu D., Okabe M., Takaoka A., Kida H., Iwai A. (2017). *Aureobasidium pullulans* produced β-glucan is effective to enhance Kurosengoku soybean extract induced Thrombospondin-1 expression. Sci. Rep..

[19] No H., Kim J., Seo C.-R., Lee D.E., Kim J.H., Kuge T., Mori T., Kimoto H., Kim J.-K. (2021). Anti-inflammatory effects of β-1,3-1,6-glucan derived from black yeast *Aureobasidium pullulans* in RAW264.7 cells. Int. J. Biol. Macromol..

[20] Muramatsu D., Iwai A., Aoki S., Uchiyama H., Kawata K., Nakayama Y., Nikawa Y., Kusano K., Okabe M., Miyazaki T. (2012). β-Glucan derived from *Aureobasidium pullulans* is effective for the prevention of influenza in mice. PLoS ONE.

[21] Suzuki T., Kusano K., Kondo N., Nishikawa K., Kuge T., Ohno N. (2021). Biological activity of high-purity β-1,3-1,6-glucan derived from the black yeast *Aureobasidium pullulans*: A literature review. Nutrients.

[22] Zhang Y., Li Y., Xia Q., Liu L., Wu Z., Pan D. (2021). Recent advances of cereal β-glucan on immunity with gut microbiota regulation functions and its intelligent gelling application. Crit. Rev. Food Sci. Nutr..

[23] Mosmann T. (1983). Rapid colorimetric assay for cellular growth and survival: Application to proliferation and cytotoxicity assays. J. Immunol. Methods.

[24] Reunanen J., Kainulainen V., Huuskonen L., Ottman N., Belzer C., Huhtinen H., de Vos W.M., Satokari R. (2015). *Akkermansia muciniphila* adheres to enterocytes and strengthens the integrity of the epithelial cell layer. Appl. Environ. Microbiol..

[25] Thanou M.M., Kotzé A.F., Scharringhausen T., Lueßen H.L., de Boer A.G., Verhoef J.C., Junginger H.E. (2000). Effect of degree of quaternization of N-trimethyl chitosan chloride for enhanced transport of hydrophilic compounds across intestinal Caco-2 cell monolayers. J. Control. Release.

[26] Duizer E., Penninks A.H., Stenhuis W.H., Groten J.P. (1997). Comparison of permeability characteristics of the human colonic Caco-2 and rat small intestinal IEC-18 cell lines. J. Control. Release.

[27] Ménard S., Lacroix-Lamandé S., Ehrhardt K., Yan J., Grassl G., Wiedemann A. (2022). Cross-talk between the intestinal epithelium and *Salmonella* Typhimurium. Front. Microbiol..

[28] Kuda T., Kosaka M., Hirano S., Kawahara M., Sato M., Kaneshima T., Nishizawa M., Takahashi H., Kimura B. (2015). Effect of sodium-alginate and laminaran on *Salmonella* Typhimurium infection in human enterocyte-like HT-29-Luc cells and BALB/c mice. Carbohydr. Polym..

[29] Shao Y., Guo Y., Wang Z. (2013). β-1,3/1,6-Glucan alleviated intestinal mucosal barrier impairment of broiler chickens challenged with *Salmonella enterica* serovar Typhimurium. Poult. Sci..

[30] Martel J., Chang S.H., Ko Y.F., Hwang T.L., Young J.D., Ojcius D.M. (2022). Gut barrier disruption and chronic disease. Trends Endocrinol. Metab..

[31] Nie Y., Cao M., Wu D., Li N., Peng J., Yi S., Yang X., Zhang M., Hu G., Zhao J. (2018). KH-type splicing regulatory protein is regulated by nuclear factor-κB signaling to mediate innate immunity in Caco-2 cells infected by *Salmonella* Enteritidis. Folia Microbiol..

[32] Lépine A.F.P., de Wit N., Oosterink E., Wichers H., Mes J., de Vos P. (2018). Lactobacillus acidophilus Attenuates *Salmonella*-induced stress of epithelial cells by modulating tight-junction genes and cytokine responses. Front. Microbiol..

[33] Malago J.J., Koninkx J.F.J.G., Tooten P.C.J., Van Liere E.A., Van Dijk J.E. (2005). Anti-inflammatory properties of heat shock protein 70 and butyrate on *Salmonella*-induced interleukin-8 secretion in enterocyte-like Caco-2 cells. Clin. Exp. Immunol..

[34] Malago J.J., Tooten P.C., Koninkx J.F. (2010). Anti-inflammatory properties of probiotic bacteria on *Salmonella*-induced IL-8 synthesis in enterocyte-like Caco-2 cells. Benef. Microbes.

[35] Cao Y., Sun Y., Zou S., Duan B., Sun M., Xu X. (2018). Yeast β-glucan suppresses the chronic inflammation and improves the microenvironment in adipose tissues of ob/ob Mice. J. Agric. Food Chem..

[36] Walachowski S., Tabouret G., Fabre M., Foucras G. (2017). Molecular analysis of a short-term model of β-glucans-trained immunity highlights the accessory contribution of GM-CSF in priming mouse macrophages response. Front. Immunol..

[37] Kawata K., Iwai A., Muramatsu D., Aoki S., Uchiyama H., Okabe M., Hayakawa S., Takaoka A., Miyazaki T. (2015). Stimulation of macrophages with the β-glucan produced by *Aureobasidium pullulans* promotes the secretion of tumor necrosis factor-related apoptosis inducing ligand (TRAIL). PLoS ONE.

[38] Tada R., Yoshikawa M., Kuge T., Tanioka A., Ishibashi K., Adachi Y., Tsubaki K., Ohno N. (2009). A highly branched 1,3-beta-D-glucan extracted from *Aureobasidium pullulans* induces cytokine production in DBA/2 mouse-derived splenocytes. Int. Immunopharmacol..

[39] Tada R., Yoshikawa M., Ikeda F., Adachi Y., Kato Y., Kuge T., Tanioka A., Ishibashi K.-I., Tsubaki K., Ohno N. (2011). Induction of IFN-γ by a highly branched 1,3-β-d-glucan from *Aureobasidium pullulans* in mouse-derived splenocytes via dectin-1-independent pathways. Biochem. Biophys. Res. Commun..

[40] Tada R., Yoshikawa M., Kuge T., Tanioka A., Ishibashi K., Adachi Y., Tsubaki K., Ohno N. (2011). Granulocyte macrophage colony-stimulating factor is required for cytokine induction by a highly 6-branched 1,3-β-D-glucan from *Aureobasidium pullulans* in mouse-derived splenocytes. Immunopharmacol. Immunotoxicol..

[41] Raupach B., Peuschel K., Monack A. (2006). Caspase-1-mediated activation of interleukin-1beta (IL-1beta) and IL-18 contributes to innate immune defenses against *Salmonella enterica* serovar Typhimurium infection. Infect. Immun..

[42] Billiet T., Rutgeerts P., Ferrante M., Van S. (2014). Targeting TNF-α for the treatment of inflammatory bowel disease. Expert. Opin. Biol. Ther..

[43] Breese J., Michie A., Nicholls W., Murch H., Williams B., Domizio P., Walker A., Macdonald T. (1994). Tumor necrosis factor α-producing cells in the intestinal mucosa of children with inflammatory bowel disease. Gastroenterology.

[44] Wang Z., Ma K., Fujino M., Kusano K., Yi S.-Q., Iwai A., Li X.-K. (2021). The effects of oral administration of *Aureobasidium pullulans*-cultured fluid containing β-glucan on concanavalin A injected mice. Heliyon.

[45] Hayashi N., Shoubayashi Y., Kondo N., Fukudome K. (2019). Hydrothermal processing of β-glucan from *Aureobasidium pullulans* produces a low molecular weight reagent that regulates inflammatory responses induced by TLR ligands. Biochem. Biophs. Res. Commun..

[46] Elmaksoud H.A.A., Motawea M.H., Desoky A.A., Elharrif M.G., Ibrahimi A. (2021). Hydroxytyrosol alleviate intestinal inflammation, oxidative stress and apoptosis resulted in ulcerative colitis. Biomed. Pharmacother..

[47] Rao R. (2008). Oxidative stress-induced disruption of epithelial and endothelial tight junctions. FBL.

[48] Ookawara T., Imazeki N., Matsubara O., Kizaki T., Oh-Ishi S., Nakao C., Sato Y., Ohno H. (1998). Tissue distribution of immunoreactive mouse extracellular superoxide dismutase. Am. J. Physiol..

[49] Sottero B., Rossin D., Poli G., Biasi F. (2018). Lipid oxidation products in the pathogenesis of inflammation-related gut diseases. Curr. Med. Chem..

[50] Xiao X., Zhou Y., Tan C., Bai J., Zhu Y., Zhang J., Zhou X., Zhao Y. (2021). Barley β-glucan resist oxidative stress of Caenorhabditis elegans via daf-2/daf-16 pathway. Int. J. Biol. Macromol..

